# Effects of 12 Weeks of Interval Block Resistance Training Versus Circuit Resistance Training on Body Composition, Performance, and Autonomic Recovery in Adults: Randomized Controlled Trial

**DOI:** 10.3390/jfmk10020195

**Published:** 2025-05-28

**Authors:** Héctor Fuentes-Barría, Raúl Aguilera-Eguía, Juan Maureira-Sánchez, Miguel Alarcón-Rivera, Victor Garrido-Osorio, Olga Patrica López-Soto, Juan Alberto Aristizábal-Hoyos, Lissé Angarita-Davila, Diana Rojas-Gómez, Valmore Bermudez, Cherie Flores-Fernández, Ángel Roco-Videla, Jorge Enrique González-Casanova, Sebastian Urbano-Cerda, Dan Iulian Alexe

**Affiliations:** 1 Vicerrectoría de Investigación e Innovación, Universidad Arturo Prat, Iquique 1110939, Chile; 2 Departamento de Salud Pública, Facultad de Medicina, Universidad Católica de la Santísima Concepción, Concepción 3349001, Chile; raguilerae@ucsc.cl; 3 Programa de Doctorado en Educación, Facultad de Educación, Universidad Bernardo O’Higgins, Santiago 7550000, Chile; juan.maureira74@gmail.com; 4 Escuela de Ciencias del Deporte y Actividad Física, Facultad de Salud, Universidad Santo Tomás, Talca 3460000, Chile; mrivera3@santotomas.cl; 5 Facultad de Medicina, Universidad Católica del Maule, Talca 3460000, Chile; 6 Instituto de Ciencias de la Salud, Universidad de O`Higgins, Rancagua 2820000, Chile; vgarrido@fitbike.cl; 7 Departamento de Salud Oral, Facultad de Salud, Universidad Autónoma de Manizales, Caldas 170008, Colombia; sonrie@autonoma.edu.co (O.P.L.-S.); jaristi@autonoma.edu.co (J.A.A.-H.); 8 Escuela de Nutrición y Dietética, Facultad de Medicina, Universidad Andres Bello, Concepción 3349001, Chile; lisse.angarita@unab.cl; 9 Escuela de Nutrición y Dietética, Facultad de Medicina, Universidad Andres Bello, Santiago 7550000, Chile; diana.rojas@unab.cl; 10 Facultad de Ciencias de la Salud, Universidad Simón Bolívar, Barranquilla 080022, Colombia; valmore.bermudez@unisimon.edu.co; 11 Departamento Gestión de la Información, Universidad Tecnológica Metropolitana, Santiago 7550000, Chile; cflores@utem.cl; 12 Programa de Magíster en Ciencias Químico Biológicas, Facultad de Ciencias de la Salud, Universidad Bernardo O’Higgins, Santiago 7550000, Chile; angel.roco.videla@gmail.com; 13 Facultad de Ciencias de la Salud, Instituto de Ciencias Biomédicas, Universidad Autónoma de Chile, Santiago 8910060, Chile; jorge.gonzalez@uautonoma.cl; 14 Programa Magíster en Ciencias de la Actividad Física y del Deporte Aplicadas al Entrenamiento, Rehabilitación y Reintegro Deportivo, Facultad de Salud, Universidad Santo Tomás, Santiago 7550000, Chile; sebaurbano114@gmail.com; 15 Department of Physical and Occupational Therapy, “Vasile Alecsandri” University of Bacău, 600115 Bacău, Romania; alexedaniulian@ub.ro

**Keywords:** resistance training, circuit-based exercise, body composition, muscle strength, physical fitness, muscle fatigue

## Abstract

**Objectives:** Interval block resistance training (IBRT) and circuit resistance training (CRT) are periodization models aimed at enhancing neuromuscular and metabolic adaptations. This study aims to evaluate the effects of a 12-week IBRT program compared to CRT on body composition, muscle strength, speed, functional capacity, and autonomic recovery in young Chilean adults. **Methods:** A randomized, parallel, double-blind study was conducted with 30 participants assigned to IBRT (n = 15) or CRT (n = 15). Assessments included body mass index (BMI), waist circumference, right-hand grip strength, the running anaerobic sprint test (RAST), the 6 min walk test (6 MWT), and heart rate variability (HRV) indices: low-frequency to high-frequency ratio (LF/HF) and root mean square of successive differences (RMSSD, a time-domain HRV metric reflecting parasympathetic activity). Statistical analyses included *t*-tests and ANCOVA. **Results:** Groups were similar in age (IBRT: 25.2 ± 3.19; CRT: 23.27 ± 3.69, *p* = 0.14) and BMI (IBRT: 21.56 ± 2.22; CRT: 22.36 ± 1.70 kg/m^2^, *p* = 0.40). Both groups improved significantly in waist circumference (IBRT: −1.85%; CRT: −2.37%), grip strength (IBRT: +5.47%; CRT: +4.02%), RAST (IBRT: −2.67%; CRT: −1.04%), 6 MWT (IBRT: +4.53%; CRT: +2.17%), LF/HF (IBRT: −11.43%; CRT: −5.11%), and RMSSD (IBRT: +5.36%; CRT: +3.81%) (all *p* ≤ 0.01). IBRT produced significantly greater gains in 6 MWT (B = 19.51, 95% CI: 0.79 to 38.23, *p* = 0.04). **Conclusions:** Both IBRT and CRT effectively improved body composition, muscle strength, speed, functional capacity, and autonomic recovery. However, IBRT demonstrated a superior effect on aerobic capacity.

## 1. Introduction

The organization of resistance training is based on three fundamental levels: planning, periodization, and programming. Planning establishes long-term objectives and the general distribution of training cycles [1]. Periodization structures these cycles to optimize adaptations over time, using strategies such as block periodization or undulating models. Finally, programming details the specific distribution of training within each session, regulating variables such as intensity, volume, and density to achieve targeted physiological responses [2,3,4].

Following this framework, the training protocol used in this study follows a structured programming strategy, which integrates elements of block periodization but does not align with or strictly follow Verkhoshansky’s block periodization model. Although it was initially developed for sports with high demands on strength and speed, over time, it has also been adopted in endurance disciplines [2,3]. In these sports, modern competitions require optimizing both technical aspects and preexisting speed manifestations to enhance performance and increase the chances of sporting success [3].

The training model in this study follows an interval block resistance training (IBRT) approach, which structures resistance exercises into focused work segments within each session to enhance physiological adaptations. While it shares some similarities with block periodization, it does not adhere to the traditional block periodization framework, which involves sequential phases of accumulation, transmutation, and realization. Instead, the training load is distributed within short, high-intensity intervals, where each segment maintains a strategic variation in effort distribution to achieve specific training objectives [4]. This structured programming approach leverages cumulative training effects through alternating intensities, optimizing both neuromuscular and metabolic responses. As a result, it induces progressive adaptations in morphological and metabolic components, enhancing athletic performance [5,6].

In this context, it has been shown that block periodization can generate beneficial effects on both body composition and manifestations of speed, strength, and muscular endurance related to sports performance [7,8,9]. IBRT promotes a reduction in body fat and an increase in muscle tissue, modulated by the hypertrophy process associated with high-intensity progressive training, which can maintain tissue adaptations even after three weeks of inactivity [10]. Muscle hypertrophy occurs through the activation of signaling pathways such as mTOR and PI3K. The mTOR pathway is primarily responsible for anabolic functions related to protein synthesis and muscle fiber growth, while the PI3K pathway regulates muscle protein turnover by inhibiting protein degradation processes [11]. During the hypertrophic process, the availability of energy substrates conditions the activation of type II muscle fibers (fast contraction), which are responsible for generating high levels of strength and are highly sensitive to overreaching and mechanical tension [12].

Similarly, IBRT has been widely demonstrated to focus stimuli on the neuromuscular activation process, which enhances motor unit recruitment and, consequently, the capacity to generate muscular power and speed [8,13,14,15]. In this context, the repetitive stimuli typical of IBRT distributions lead to increased efficiency in the central nervous system’s coordination of motor unit activation, resulting in improvements in maximal strength [16]. This enhancement in motor unit recruitment, also for a greater expression of the rate of force development, is a key element in sports movements with high muscle power components [15].

At the metabolic level, it is known that IBRT improves both aerobic and anaerobic capacity, commonly associated with sports that involve both strength and high endurance components, where improvements in aerobic power contribute to better performance in submaximal efforts [2,3]. From a bioenergetic perspective, the IBRT model primarily relies on the phosphagen system (ATP-PCr) and anaerobic glycolysis during short, intense efforts, while the aerobic system contributes during rest intervals and recovery periods. This strategic combination enhances metabolic flexibility and improves energy efficiency across different intensities. In endurance-dominant sports, the modulation of mitochondrial biogenesis by the transcription factor PGC-1α, along with peroxisome proliferation, enhances substrate mobilization and the oxidative process, directly impacting physical performance [17]. Additionally, the high intensity of the load helps optimize fatty acid oxidation and glucose absorption in muscle cells through the activation of AMPK, which, in turn, modulates muscle architecture [17]. This approach helps avoid overreaching, enhancing physiological adaptations that sustain high performance while reducing the risk of sports injuries [17,18,19].

In this regard, IBRT has undergone multiple variations and alternatives within contemporary models, with the popularization of undulating load distribution being a complementary response that provides an effective model for mimicking the oscillations associated with the general adaptation syndrome and supercompensation. By organizing exercises in circuits and varying the load within short cycles, this approach helps prevent performance plateaus and induces greater adaptation. As a result, it maximizes gains in key parameters such as body composition, strength, and muscular endurance, thus enhancing overall athletic performance [20,21,22,23].

Recent evidence suggests that undulating periodization has a significant impact on body composition, particularly by promoting fat reduction and muscle mass gains. These effects are driven by the combination of metabolic stimuli associated with hypertrophic processes, where alternating load volumes induce an anabolic response, optimizing body restructuring through protein synthesis and type II fiber recruitment [24,25,26]. Regarding neuromuscular adaptations, motor unit recruitment capacity plays a crucial role in sustained increases in maximal strength, which, in turn, improves explosive strength in short time frames. This translates into improvements in muscle power, acceleration, and movement speed, particularly during the initial phases of a training program [27,28,29], where the so-called CRT shows positive effects on cardiorespiratory function, muscle strength, body composition, and glycemic control [30,31,32,33,34].

The resulting neuromuscular efficiency has a direct impact on movement mechanics, leading to a greater economy in sports-specific gestures. These adaptations contribute to enhanced functional capacity, as the intermittent demands of endurance training align more effectively with cardiovascular adaptations, such as cardiovascular remodeling at the capillary density level and the expansion of stroke volume [24,35,36]. Furthermore, the inherent variability of stimuli in undulating periodization circuit resistance training (CRT) helps minimize fatigue and prevent overreaching by alternating the involvement of different muscle systems. This strategic variation optimizes recovery by mitigating metabolic stress, thus promoting long-term performance sustainability [37,38,39,40,41].

While block and undulating periodization models have demonstrated various benefits in strength and endurance sports, their comparative effects on overall physical performance have been primarily studied in adolescents and older adults, with young adults being a scarcely addressed population [14,42,43,44,45,46]. Given the potential of both approaches to influence key parameters such as body composition, muscle strength, functional capacity, sprint performance, and recovery dynamics, further research is needed to determine their relative efficacy. To address this gap, the present study aims to evaluate the effects of a 12-week IBRT program compared to CRT on body composition, muscle strength, speed, functional capacity, and autonomic recovery in young Chilean adults.

## 2. Materials and Methods

### 2.1. Design

A controlled, parallel, and randomized double-blind study was conducted in accordance with the Consolidated Standards of Reporting Trials (CONSORT) checklist [47]. The study protocol and informed consent were approved by the Ethics Committee of the Central University of Chile (Protocol Code: 02/2025; Approval Date: 31 January 2025). The trial was subsequently registered with the International Standard Randomised Controlled Trial Number (ISRCTN) (Protocol Code: ISRCTN17853333; DOI: 10.1186/ISRCTN17853333; Registration Date: 4 February 2025), in accordance with the Declaration of Helsinki [48].

### 2.2. Eligibility

The participants were invited to the Municipal Stadium, located in the commune of Pirque in the Metropolitan Region of Chile, between 3 February and 25 April 2025. At this venue, a sports science specialist, certified as a level I coach by World Athletics, assessed the participant eligibility selection. Each participant received a brief written description of the study, including its objectives, along with an informed consent form. Before starting the intervention, volunteers were required to meet the following eligibility criteria.

#### 2.2.1. Inclusion Criteria

- Aged between 18 and 30 years and classified as a physically active adult.
- Refrain from engaging in moderate or intense physical activity during the 48 h prior to each session to prevent interference with acute training responses.
- Attend all scheduled training sessions punctually throughout the 12-week intervention.
- Read, understand, and sign the informed consent form before undergoing evaluations.

#### 2.2.2. Exclusion Criteria

- Diagnosis of conditions such as hypertension, type 2 diabetes, coronary artery disease, or other cardiovascular, metabolic disorders, or inability to exercise due to injury.
- Body mass index within the overweight or obese range, along with a waist circumference exceeding the high cardiometabolic risk threshold of 88 cm for the Chilean adult population.
- Hand grip strength below the threshold 50 kg was considered a risk of muscle weakness in the classification for the Chilean adult population
- Performance below the 644 m reference threshold for reduced functional capacity in the Chilean adult population.
- Participation in another training program during the intervention, which could interfere with the study protocol response.

**Figure 1:**
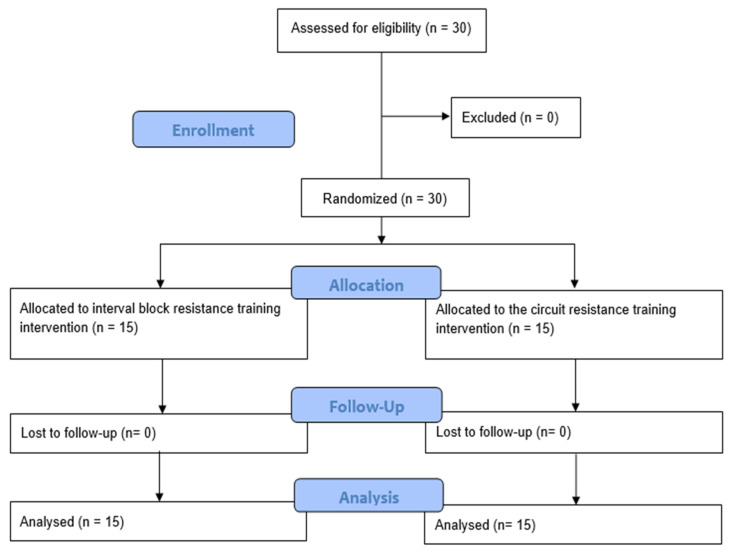
Enrollment, allocation, follow-up, and analysis of participants.

Figure 1 presents the flowchart outlining the recruitment, allocation, follow-up, and analysis process for the study participants. A total of 30 adult men who participated in endurance sports such as running and amateur triathlon were included, with 15 assigned to the experimental group and 15 to the control group.

### 2.3. Training Protocols

Table 1 shows how the participants underwent a 12-week resistance training program with a frequency of 3 sessions per week (Monday, Wednesday, and Friday), each lasting 48 and 57 min, based on a resistance training program previously used with physically active young adults [49,50,51]. Each session was structured into three phases: warm-up, main phase, and cool-down.

Table 2 presents the volume, intensity, and density of the training load, which were identical for both the experimental group and control group. The only difference between the groups was the exercise distribution.

Specifically, a systematic variation in the exercise order was applied across the 12 weeks, structured into four mesocycles of three weeks each. This traditional structure, based on undulating periodization models, allowed for a progressive and goal-oriented organization of training. The first mesocycle focused on anatomical and technical adaptation; the second mesocycle aimed to develop muscular endurance; the third mesocycle sought to increase training load and stimulate the adaptation syndrome; and the fourth mesocycle aimed to transfer the accumulated adaptations toward physical performance. This progression followed a non-linear periodization model based on relative training intensity fluctuations between sessions [52].

Regarding workload volume and exercise selection, both groups performed the same six exercises: Push-up, Mountain climber, Squat, Jumping Jack, Burpees, and Skipping. Each training session consisted of 6 sets of 6 repetitions per exercise. However, the structure differed between groups [49,50,51]:

- The experimental group followed an IBRT protocol, where each set consisted of a different exercise in the session (Set 1: Push-up, Set 2: Mountain climber, Set 3: Squat, Set 4: Jumping Jack, Set 5: Burpees, Set 6: Skipping).
- The control group followed a CRT protocol, in which all six exercises were performed sequentially within each set, and this same circuit was repeated across all sets.

The six exercises performed by both intervention groups were selected based on previous protocols applied to Chilean adults in similar contexts [49,50,51]. All exercises were chosen for their functional nature and their emphasis on developing muscular strength through multi-joint movements with high metabolic demand. These characteristics facilitated the standardization of an effective training protocol without the need for auxiliary external load equipment, such as weight plates [53,54].

The intensity of the training was initially estimated indirectly using the maximum heart rate calculated using the Tanaka equation adapted from Karvonen’s proposals [55,56]:

Maximum Heart Rate = 220 − age

Regarding resting heart rate, it was individually monitored during each session using a Polar^®^ Vantage V2 watch and a Polar^®^ H10 heart rate monitor (Polar Electro Oy, Kempele, Finland) [57,58]. Measurements were taken with participants lying in a supine position for 5 min. The relative intensity of each session was assessed based on the percentage of the estimated maximum heart rate, calculated using the Tanaka equation [54].

The entire training protocol was executed under the supervision of a certified sports science specialist, who was also responsible for ensuring that both IBRT and CRT participants were working consistently ~75% of their estimated maximum heart rate during each session, thereby maximizing the intensity of interval training to achieve physiological adaptation as previously recommended [59].

It is worth mentioning that both before and after this exercise program, the following evaluations were carried out.

#### 2.3.1. Level of Physical Activity

The level of physical activity was assessed by a sports science specialist using the short version of the International Physical Activity Questionnaire (IPAQ-short), which consists of seven questions that evaluate the frequency and intensity of physical activity during the last seven days. This tool has been validated in the adult Chilean population, and physical activity levels were categorized according to the Metabolic Equivalent of Task (MET) into low (<599), moderate (600–1499), and high (≥1500) [60].

Although the IPAQ has known limitations, especially when compared to objective measures such as accelerometry, it was selected due to its wide acceptance by the WHO and the scientific community, its validated use in Chile, and its practical applicability [60,61,62,63]. Given the logistical limitations of using accelerometers in this study, it allows us to classify people as physically active or inactive based on our study focus.

#### 2.3.2. Body Composition

The height of the participants was recorded using a portable Cescorf height rod (São Paulo, Brazil) with a maximum length of 300 cm, validated for anthropometric purposes by the International Society for the Advancement of Kinanthropometry (ISAK) [64,65]. Body weight was measured using a SECA model 803 digital scale, whose characteristics comply with ISAK recommendations for a range of 0 to 150 kg with a precision of 100 gr [65]. These measurements were then used to calculate the body mass index using the following equation [66]:

Body mass index = body weight (kg)/height (m)^2^

Waist circumference was measured using a Cescorf metal anthropometric tape (São Paulo, Brazil), which has an average resolution of ±1 mm, is 6 mm wide, and 200 cm long. This tape is validated for anthropometric purposes [64,65]. For this measurement, the participant was instructed to stand with their feet together with a relaxed abdomen. The tape was placed horizontally just above the iliac crest, encircling the abdomen at its narrowest point, which typically coincides with the level of the umbilicus. The waist circumference was recorded after exhalation. In Chilean men, a measurement ≥88 cm is considered indicative of obesity and a high cardiometabolic risk [67].

Both weight, height, and waist circumference measurements were performed by an ISAK level II-certified instructor, whose measurement error has been estimated by the literature at 5% [65].

#### 2.3.3. Hand Grip Strength

This test was evaluated by a sports science specialist, who began the test by seating the participant in a chair with a backrest, with the elbow flexed at 90° and the wrist in a neutral or slightly extended position (0° to 30°) to allow for a proper gripping action with the palm of the hand. The objective of the test is to exert maximum force for 3 s, recording the highest value from three attempts, each separated by a 1 min recovery period for both the left and right hands. A result of ≥50 kg is considered an indicator of good health in the Chilean population [68].

This test uses a Camry^®^ EH101 digital dynamometer (Zhongshan, China), with a maximum capacity of 90 kg and an accuracy of 0.1 kg. It has demonstrated a high interclass correlation coefficient (ICC = 0.97) when compared to the JAMAR^®^ J00105 hydraulic dynamometer (Bolingbrook, IL, USA). [69]. This has been validated as a reliable alternative for assessing hand grip strength and nutritional status [70,71], being calibrated according to the manufacturer’s recommendation regarding the adjustment of each participant’s grip.

#### 2.3.4. Running Anaerobic Sprint Test

This test evaluated 6 repeated sprints of 35 m separated with a recovery interval of 10 s between each attempt, recording the best time used to complete 35 linear m. This test is widely used in sports that demand high levels of muscular strength, explosiveness, and sprinting ability, with performance values between 4.61 and 5.74 s considered normal in the adult population [72].

The evaluation was carried out by a sports science specialist, who measured a 35 m straight stretch on a 400 m athletics track using a Crownman^®^ CM-OM1318 folding digital odometer with a 320 mm wheel (Taiwan, China). This device can measure distances of up to 10,000 m with an accuracy of 0.1 m. Performance was recorded based on the time recorded by a Casio^®^ HS-3V-1B unisex digital stopwatch (Shibuya, Tokio, Japan).

#### 2.3.5. Six Min Walking Test

This test was designed to determine the maximum distance a person can walk at a steady pace unaided for 6 min over a 30 m round trip. The result of this test is widely used to assess functional capacity both in healthy individuals and in populations with respiratory and cardiovascular diseases, with values <644 m being considered as a prognostic factor for survival and functional decline in the healthy Chilean population aged 20–80 years [73].

The evaluation was carried out by a sports science specialist on a 400 m running track; the distance was determined using a Crownman^®^ CM-OM1318 folding digital odometer with a 320 mm wheel (Taiwan, China). This device can measure distances of up to 10,000 m with an accuracy of 0.1 m. Elapsed time was monitored using a Casio^®^ HS-3V-1B unisex digital stopwatch (Shibuya, Tokio, Japan).

#### 2.3.6. Autonomic Recovery

The assessment was conducted by a sports science specialist in a quiet environment using the Polar^®^ H10 heart rate monitor in conjunction with the Polar^®^ Vantage V2 watch (both from Polar Electro Oy, Kempele, Finland) to record RR intervals. These devices have been widely validated for use in recording heart rate variability (HRV) under both resting and exercise conditions [57,58].

This setup enabled the application of a 4 min orthostatic test prior to the first weekly training session to evaluate autonomic recovery capacity through the root mean square of successive differences (RMSSD), with the result being directly extracted from the Polar^®^ Vantage V2 watch (Polar Electro Oy, Kempele, Finland) [57,58].

Autonomic balance was assessed at the beginning of each training week by analyzing the LF/HF ratio, obtained from a short-term (5 min) resting HRV recording using the Polar^®^ H10 monitor while participants were lying in a supine position [52,53,74]. The LF/HF ratio data were exported to the ELITE HRV smartphone app (Asheville, NC, USA) and subsequently analyzed with Kubios HRV software version 5.1 for Windows (Kuopio, Finland) [75,76].

The RMSSD and LF/HF ratio were used to assess autonomic recovery status. Specifically, an LF/HF ratio between 1.5 and 2 is considered indicative of normal autonomic balance, while RMSSD values ranging from 30 to 50 ms reflect moderate HRV in healthy adults [77,78]. In this context, an increase in the LF/HF ratio alongside a decrease in RMSSD beyond these reference ranges suggests reduced HRV, indicating a diminished capacity for recovery and adaptation to training loads. Conversely, a lower LF/HF ratio combined with a higher RMSSD reflects enhanced autonomic recovery and greater adaptability to exercise-induced stress [79]. However, the interpretation of these dynamics may vary depending on factors such as age, physical activity level, overall health status, and stress [79].

### 2.4. Randomization

The randomization sequence was generated using an online random sequence generator (https://www.randomizer.org/). This process was carried out in a stratified manner through an ordered 1:1 allocation, ensuring that the sizes of each group or block were equivalent. Participants were randomly assigned using a simple, coded randomization method, which helps to ensure unbiased group allocation and maintain the integrity of the study’s design.

### 2.5. Blinding

This study employed a double-blind design to minimize bias in the assessment of training effects. Participants were unaware of the specific study hypothesis and were informed that both protocols were designed to improve strength performance. They were also instructed not to discuss their group assignment with other participants. Additionally, the assessors were blinded to the group assignments, ensuring that evaluations were conducted in a standardized manner to make sure data collection was independent of the training type. Similarly, the trainer overseeing the sessions was different from the assessor to avoid potential biases. Furthermore, the training sessions were conducted separately to prevent cross-group observations.

### 2.6. Sample Size and Statistical Power

The study included a sample of 30 participants, evenly distributed into two groups of 15 individuals each. Participants were selected through non-probabilistic sampling based on practical criteria that ensured adequate levels of physical activity and the absence of chronic diseases. Although the sample size was limited, it was determined according to logistical constraints and ethical considerations related to the recruitment process.

Consequently, a post hoc two-tailed power analysis was conducted using G*Power software version 3.1.9.7 for Windows (Dusseldorf, Germany), with a significance level of 0.05 and a large effect size (r = 0.8). The estimated statistical power was 56%.

Despite this limitation, the randomized, controlled, and double-blind experimental design, along with the baseline homogeneity between groups (*p* > 0.05), helped minimize variability. This preserved the internal validity of the study and allowed for a more accurate interpretation of the results, even though the estimated power was below the recommended 80% threshold, with the sample size required to reach this threshold being 52 participants [80,81].

### 2.7. Data Analysis

The data were analyzed using IBM SPSS Statistics version 27.0 for Windows (Armonk, NY, USA). The Shapiro–Wilk test was used to assess the normality of the data distribution, while the Levene test evaluated the homogeneity of variances, confirming a normal distribution of the variables. For the descriptive analysis, measures of central tendency mean ($\bar{X}$), dispersion standard deviation (SD), and percentage variation (%) were calculated to summarize performance changes across variables. In the inferential analysis, an independent samples *t*-test was used to compare values between groups IBRT and CRT. Effect sizes were reported using Cohen’s d, categorized as small (≥0.2), moderate (≥0.5), or large (≥0.8) [80,81]. Additionally, a univariate ANCOVA analysis was conducted exclusively for the 6 min walk test, as it showed significant differences between the groups. In this analysis, the post-test was considered the dependent variable, the pre-test as the covariate, and the IBRT and CRT groups as fixed factors. The results of the analysis were expressed using the beta unstandardized coefficient (B), 95% confidence intervals (CI), standard error, and a bi-lateral significance or *p*-value of 0.05, which was also applied to the *t*-test.

## 3. Results

The intervention included 30 physically active young adults with normal weight and low cardiometabolic risk. Participants were assigned to an experimental group (n = 15), with a mean age of 25.2 ± 3.19 years, and a control group (n = 15), with a mean age of 23.27 ± 3.69 years.

Table 3 presents the indicators of body composition and athletic performance for both the experimental group and the control group before the intervention. No statistically significant differences were observed between the groups across any of the assessed variables (*p* > 0.05), indicating a homogeneous distribution of participants prior to the intervention.

Regarding body composition, both groups exhibited similar values for weight, body mass index, and waist circumference. In terms of athletic performance, hand grip strength measurements showed a slight advantage in favor of the control group, although this difference did not reach statistical significance. Likewise, performance in the running anaerobic sprint test and the 6 min walk test were comparable between groups.

With respect to HRV indicators, the LF/HF ratio and RMSSD values were similar between groups, reflecting an autonomic balance prior to the intervention. These findings suggest that both groups started the study with equivalent baseline characteristics, ensuring that any observed effects can be attributed to the training protocol rather than preexisting differences.

Table 4 presents the changes observed in body composition and physical performance parameters before and after the intervention in the experimental group (n = 15). Significant improvements were noted in waist circumference reduction (*p* ≤ 0.01, % = 1.85), increased dominant hand grip strength (*p* ≤ 0.01, % = 5.47), sprint speed (*p* ≤ 0.01, % = 2.67), functional capacity (*p* ≤ 0.01, % = 4.53), LF/HF ratio (*p* ≤ 0.01, % = 11.43), and RMSSD (*p* ≤ 0.01, % = 5.36).

Table 5 shows the changes in body composition and physical performance parameters in the control group (n = 15) before and after the intervention. Significant improvements were observed in waist circumference reduction (*p* ≤ 0.01, % = 2.37), increased dominant hand grip strength (*p* ≤ 0.01, % = 4.02), running anaerobic sprint test (*p* = 0.010, % = 1.04), functional capacity as measured by the 6 min walking test (*p* ≤ 0.01, % = 2.17), LF/HF ratio (*p* ≤ 0.01, % = 5.11), and RMSSD (*p* ≤ 0.01, % = 3.81).

Table 6 presents indicators of body composition and athletic performance after the intervention of both the experimental group (n = 15) and control group (n = 15). A significant improvement was observed in the 6 min walking test (*p* = 0.04, % = 4.53). The results suggest that the experimental intervention led to functional improvements but had little impact on body composition or strength outcomes compared to the control group.

Table 7 presents the ANCOVA analysis, which revealed that before and after the intervention, both for the experimental group (n = 15) and the control group (n = 15), the pre-test score was a significant predictor of post-test performance (B = 0.81, *p* < 0.001), indicating a strong relationship between initial and final walking distance. After adjusting for baseline score differences, a significant difference was observed between the groups (*p* = 0.04), with the CRT group showing less improvement in walking distance compared to the IBRT group. Specifically, the adjusted difference between the groups was 19.51 m (B = 19.51, 95% CI: 0.79 to 38.23). The model explained 67% of the variability in post-test results (Adjusted R^2^ = 0.67), indicating a good fit and further supporting the effectiveness of IBRT in improving walking performance over 12 weeks.

## 4. Discussion

The present study evaluated the effects of a 12-week interval IBRT program compared to CRT on body composition, muscle strength, speed, functional capacity, and autonomic recovery in young physically active adults. Both training modalities elicited significant improvements in waist circumference, right-hand grip strength, running anaerobic sprint test performance, 6 min walk test distance, HRV markers such as RMSSD, and autonomic balance (LF/HF ratio). However, the IBRT group demonstrated a superior improvement in aerobic endurance as reflected in the 6 min walking test, supporting the notion that concentrated high-intensity training segments enhance neuromuscular and metabolic adaptations more effectively than continuous circuits.

The reductions in waist circumference observed in both groups are consistent with prior research demonstrating the effectiveness of high-intensity resistance training in reducing central adiposity [82,83]. This effect likely results from the increased metabolic demand and elevated post-exercise oxygen consumption associated with resistance training, which promotes lipid oxidation, particularly in visceral fat, which is highly sensitive to catecholamine-induced lipolysis [84,85]. Nevertheless, the absence of significant changes in the body mass index suggests that fat loss was counterbalanced by the preservation or slight increase in lean mass, a common outcome in resistance training studies due to muscle hypertrophy processes mediated by mTOR activation [11,86,87,88,89]. Similar findings have been reported in studies comparing block and undulating periodization, where improvements in body composition occurred without substantial weight changes [43,45]. These results highlight the importance of evaluating detailed body composition changes rather than relying solely on BMI when assessing the effects of resistance training.

Regarding muscle strength, both groups exhibited significant increases in right-hand grip strength, with no significant differences between IBRT and CRT. This aligns with previous research showing that both training modalities can enhance neuromuscular recruitment and strength [90,91]. However, the greater improvements in the dominant hand may be due to asymmetries in daily usage and motor unit recruitment, a phenomenon well documented in hand dominance studies [92,93]. The lack of significant differences between IBRT and CRT in grip strength gains suggests that both training models provided sufficient mechanical load to induce neuromuscular adaptations, despite their structural differences in exercise organization.

Improvements in sprint performance observed in both groups can be attributed to neuromuscular adaptations that enhance the rate of force development, stride efficiency, and muscle power, as previously reported in studies on resistance training and sprint performance [13,94,95,96,97,98,99]. Notably, no significant difference was observed between groups, suggesting that while IBRT focuses on high-intensity segments, CRT’s continuous workload distribution may have provided a similar anaerobic stimulus, leading to comparable enhancements in speed-related performance [95,96,97]. This finding supports the notion that resistance training, regardless of periodization model, can elicit improvements in short-duration explosive movements when appropriately structured.

The superior improvement in the 6 min walking test observed in the IBRT group compared to CRT suggests that the concentrated load distribution of IBRT may be more effective in enhancing aerobic endurance and movement efficiency. Previous studies have indicated that block periodization models can optimize neuromuscular recruitment, metabolic efficiency, and cardiovascular adaptations, leading to improved submaximal exercise performance [100,101,102,103,104,105]. The observed enhancement in the IBRT group may be attributed to an improved ability to sustain higher force outputs with reduced energy expenditure, likely due to increased mitochondrial biogenesis mediated by PGC-1α activation and improved fatty acid oxidation efficiency [103]. This finding aligns with research demonstrating that concentrated high-intensity resistance training can improve endurance performance more effectively than continuous circuit models [106,107].

Such adaptations enabled participants in the IBRT group to sustain higher walking speeds, improving their performance [108,109,110]. In contrast, CRT, with its focus on interval efforts, may explain why IBRT provided a more suitable stimulus for developing strength endurance and aerobic capacity, both essential for prolonged endurance tests [30,31,32,33,34,36,40,41].

Since optimizing recovery is crucial for performance and reducing chronic fatigue, future research should investigate the impact of different periodization models on recovery through stress biomarkers like cortisol levels, plasma creatinine, and neuromuscular fatigue [29,111].

From a molecular perspective, the observed adaptations in both training modalities may be attributed to several key cellular processes, particularly those involving muscle hypertrophy, mitochondrial biogenesis, and metabolic efficiency. High-intensity resistance training, such as IBRT, has been shown to activate the mTOR pathway, which plays a crucial role in muscle protein synthesis and hypertrophy. This activation leads to the increased synthesis of proteins necessary for muscle growth and repair, contributing to the observed gains in strength and lean mass. Additionally, the metabolic stress induced by resistance training can stimulate the PGC-1α pathway, promoting mitochondrial biogenesis and enhancing oxidative capacity. This process is particularly relevant for the IBRT group, where the concentrated high-intensity intervals likely facilitated a greater adaptation in mitochondrial function, leading to improved aerobic endurance and efficiency in fat oxidation [17,19,103].

Furthermore, the improvements in autonomic balance, as indicated by the changes in HRV markers such as RMSSD and LF/HF ratio, may reflect increased parasympathetic activity and better cardiovascular health, which are influenced by molecular mechanisms related to sympathetic and parasympathetic regulation, including adrenergic receptor sensitivity and beta-adrenergic signaling pathways. These molecular changes, resulting from both resistance training protocols, may explain the superior aerobic endurance seen in the IBRT group and the similar improvements in muscle strength and sprint performance in both groups. From a physiological perspective, the significant reductions in the LF/HF ratio and increases in RMSSD in both groups indicate an adaptive shift toward parasympathetic dominance, which has been associated with improved recovery and cardiovascular efficiency [112,113,114,115]. These autonomic improvements are in line with studies showing that resistance training enhances HRV by improving autonomic regulation, thus facilitating recovery and reducing overreaching risk [116,117,118,119].

Previous studies have shown that a 4-week period of applying the exercises incorporated in IBRT and CRT is sufficient to generate effective stimuli to improve HRV, presumably because the exercises involved imply the mobilization of large muscle groups or a high magnitude of load [49,50,51]. In this sense, both training modalities provide sufficient cardiovascular stress to induce positive adaptations in autonomic balance and adrenergic factors associated with fatty acid oxidation mechanisms, as corroborated by the significant reduction in waist circumference in both groups [120,121,122]. However, it remains unclear whether IBRT’s concentrated load structure contributes more to autonomic efficiency over a longer time frame, warranting further investigation.

## 5. Clinical and Practical Implications

The findings of this study have direct applications for strength and conditioning professionals, athletes, and individuals seeking to optimize their training programs. The significant improvements observed in both training groups suggest that both IBRT and CRT can be effective strategies for enhancing body composition, strength, and performance. However, the superior enhancement in aerobic endurance observed in the IBRT group highlights its potential for athletes engaged in sports requiring prolonged submaximal efforts, such as endurance runners, triathletes, and team sport players who rely on intermittent high-intensity bursts.

Unlike traditional models such as CRT, the proposed IBRT model is structured around high-intensity work intervals interspersed with strategically placed rest periods. This structure allows for a more precise manipulation of training variables—such as intensity, volume, and recovery—facilitating higher work quality and reduced fatigue accumulation. One of the key advantages of IBRT is its ability to simultaneously engage aerobic and anaerobic energy systems without sacrificing exercise technique or neuromuscular efficiency. Additionally, IBRT demonstrated superior improvements in autonomic recovery, as reflected by HRV measures, suggesting a more favorable adaptation of the autonomic nervous system. This is particularly relevant for athletes undergoing high training loads or individuals at risk of overreaching. In contrast, while CRT is effective for metabolic conditioning and general work capacity, its continuous nature may limit intensity and reduce recovery windows, potentially hindering performance improvements in activities requiring repeated maximal or near-maximal efforts. However, CRT remains advantageous for populations seeking time-efficient, whole-body training sessions and improvements in muscular endurance. Therefore, the selection between IBRT and CRT should consider the individual’s goals, training history, and physiological needs.

For general fitness populations, the results indicate that IBRT may be particularly beneficial for individuals seeking to improve both strength and endurance simultaneously, while CRT may be preferable for those aiming to enhance work capacity and metabolic conditioning. Additionally, the improvements in HRV and autonomic balance suggest that both training modalities can contribute to better recovery and cardiovascular health, making them viable options for populations at risk of overreaching or those with cardiometabolic concerns.

These findings also provide valuable insights for rehabilitation and clinical settings. Given that IBRT led to superior improvements in functional capacity (as measured by the 6 min walking test), it may serve as an effective approach for individuals recovering from musculoskeletal injuries or those with conditions requiring improved movement efficiency and endurance. The adaptability of IBRT in structuring training loads may allow practitioners to tailor programs based on individual recovery needs and performance goals.

In summary, this study reinforces the efficacy of resistance training for improving key physiological and performance markers, while also highlighting the advantages of IBRT in optimizing aerobic endurance. Future research should explore the long-term effects of these periodization models, their impact on different populations, and their potential for injury prevention and rehabilitation.

## 6. Limitations and Future Directions

This study presents several limitations that should be considered when interpreting the results. The relatively small sample size (n = 30) limits the generalizability of the findings, and the post hoc power analysis indicated a statistical power of 56%, suggesting the possibility of undetected differences between groups. Although strict inclusion and exclusion criteria were applied, external factors such as diet, sleep, stress, and additional physical activity were not strictly controlled, potentially influencing recovery and adaptation. While the 12-week intervention period was sufficient to observe significant improvements, it remains unclear whether these adaptations are sustainable over the long term, warranting future studies with extended follow-up periods. In terms of body composition assessment, waist circumference was used as a valid indicator of adiposity, yet more precise methods such as dual-energy X-ray absorptiometry (DXA) or segmental bioelectrical impedance analysis could provide a more detailed evaluation of fat distribution and muscle mass changes. Additionally, the study did not include physiological and hormonal biomarkers such as cortisol, creatine kinase, or lactate levels, which could offer further insights into training-induced stress and autonomic recovery dynamics. Another limitation is the specificity of the sample, consisting solely of young, physically active adults, which restricts the applicability of the findings to other populations such as older adults, women, or individuals with metabolic conditions. Future research should address these limitations by increasing sample size, implementing stricter controls over external variables, extending follow-up periods, incorporating advanced body composition analyses and physiological markers, and assessing these training models in a broader range of populations to enhance the generalizability of the results. Despite these limitations, this study provides valuable insights into the effects of interval block resistance training and circuit resistance training on physical performance and autonomic recovery, serving as a foundation for future research aimed at optimizing resistance training periodization strategies.

It is important to note that a direct test of aerobic capacity was not employed due to its physically demanding nature, which can impose excessive physiological and psychological stress on participants. Although the 6 min walk test may be considered a limitation in terms of maximal effort assessment, it was strategically selected for its balance between safety, feasibility, and its established validity in estimating functional aerobic capacity. Furthermore, this limitation was mitigated by the inclusion of complementary physical fitness assessments, such as hand grip strength for muscular force, sprint resistance for speed and muscular endurance, and HRV indices for autonomic recovery. Additionally, direct maximal tests can yield underestimated results in individuals unfamiliar with such protocols, as their performance may be limited by subjective factors such as pain tolerance and perceived exertion rather than true physiological capacity. Therefore, the chosen testing battery provides a comprehensive and pragmatic evaluation of both functional and physiological adaptations to the training interventions.

## 7. Conclusions

Both IBRT and CRT were effective in improving aerobic capacity, strength, and autonomic adaptation. However, IBRT led to greater improvements in the 6 min walking test, likely due to enhanced neuromuscular efficiency, movement economy, and metabolic adaptations. These findings suggest that IBRT may be more advantageous for endurance-based activities, whereas CRT may be better suited for improving cardiovascular fitness and metabolic conditioning. From a practical standpoint, these results highlight the importance of selecting training models based on specific performance goals. In sports requiring prolonged efforts and efficient biomechanics, IBRT may be the optimal approach, whereas CRT may benefit individuals seeking to enhance cardiovascular endurance and overall work capacity.

## Figures and Tables

**Table 1:** Resistance training program.

Phase	Duration	Type of Exercise
Warm-up	5 min	Whole-body stretching. (flexion, extension, abduction, and adduction of the shoulders, hips, knees, and ankles).
Experimental group	38–47 min	Interval block resistance training in the whole body (Push-up, Mountain climber, Squat, Jumping Jack, Burpees, and Skipping).
Control group	38–47 min	Circuit resistance training in the whole body (Push-up, Mountain climber, Squat, Jumping Jack, Burpees, and Skipping).
Cool-down	5 min	Whole-body stretching (flexion, extension, abduction, and adduction of the shoulders, hips, knees, and ankles).

**Table 2:** Twelve-week resistance training program protocol.

	Weeks											
	1	2	3	4	5	6	7	8	9	10	11	12
Maximum heart rate (%)	75	75	75	80	80	80	85	85	85	90	90	90
Set (number)	6	6	6	6	6	6	6	6	6	6	6	6
Repetitions (number)	6	6	6	6	6	6	6	6	6	6	6	6
Time for repetition (s)	30	30	30	35	35	35	40	40	40	45	45	45
Total time of working (min)	18	18	18	21	21	21	24	24	24	27	27	27
Rest between repetitions (s)	30	30	30	30	30	30	30	30	30	30	30	30
Total rest between repetitions (min)	15	15	15	15	15	15	15	15	15	15	15	15
Rest between set (min)	1	1	1	1	1	1	1	1	1	1	1	1
Total rest between set (min)	5	5	5	5	5	5	5	5	5	5	5	5
Total time (min)	38	38	38	41	41	41	44	44	44	47	47	47

**Table 3:** Indicators of body composition and athletic performance before the intervention for the experimental group (n = 15) and control group (n = 15).

Indicators	Experimental Group (X ± SD)	Control Group (X ± SD)	Variation (%)	*p*-Value	Effect Size (d)
Age (years)	25.2 ± 3.19	23.27 ± 3.69	8.29	0.14	0.56
Metabolic Equivalent of Task	2206.13 ± 397.91	2551.07 ± 386.48	15.63	0.08	0.88
Weight (kg)	63.47 ± 5.90	63.36 ± 5.54	0.17	0.96	0.02
Height (cm)	171.38 ± 5.43	168.37 ± 5.58	1.79	0.19	0.55
Body mass index (kg/m^2^)	21.56 ± 2.22	22.36 ± 1.70	3.72	0.30	0.40
Waist circumference (cm)	81.97 ± 5.07	79.25 ± 5.66	3.43	0.16	0.51
Left-hand grip (kg)	55.15 ± 4.52	56.83 ± 3.98	3.05	0.18	0.39
Right-hand grip (kg)	55.59 ± 2.91	58.04 ± 4.20	4.45	0.07	0.68
Running anaerobic sprint test (s)	4.87 ± 0.07	4.81 ± 0,10	1.23	0.09	0.67
Six min walking test (m)	697.77 ± 50.11	683.01 ± 25.78	2.17	0.25	0.37
LF/HF ratio	1.40 ± 0.18	1.37 ± 0.17	2.14	0.59	0.17
RMSSD (m/s)	49.47 ± 5.95	50.41 ± 2.90	1.90	0.52	0.20

X: mean, SD: standard deviation, LF/HF ratio: relationship between low frequency and high frequency, RMSSD: root mean square of successive differences.

**Table 4:** Indicators of body composition and athletic performance in experimental groups before and after the intervention (n = 15).

Indicators	Before (X ± SD)	After (X ± SD)	Variation (%)	*p*-Value	Effect Size (d)
Metabolic Equivalent of Task	2206.13 ± 397.91	2222.87 ± 429.26	0.76	0.41	0.04
Weight (kg)	63.47 ± 5.90	63.45 ± 5.76	0.03	0.98	0.003
Height (cm)	171.38 ± 5.43	171.38 ± 5.43	N/A	N/A	N/A
Body mass index (kg/m^2^)	21.56 ± 2.22	21.64 ± 1.97	0.37	0.71	0.04
Waist circumference (cm)	81.97 ± 5.07	80.45 ± 4.81	1.85	≤0.01	0.31
Left-hand grip (kg)	55.15 ± 4.52	56.0 ± 5.74	1.54	0.40	0.16
Right-hand grip (kg)	55.59 ± 2.91	58.63 ± 2.96	5.47	≤0.01	1.03
Running anaerobic sprint test (s)	4.87 ± 0.07	4.74 ± 0.12	2.67	≤0.01	1.30
Six min walking test (m)	697.77 ± 50.11	729.34 ± 46.85	4.53	≤0.01	0.65
LF/HF ratio	1.40 ± 0.18	1.24 ± 0.16	11.43	≤0.01	0.94
RMSSD (m/s)	49.47 ± 5.95	52.12 ± 5.71	5.36	≤0.01	0.45

X: mean, SD: standard deviation, N/A: not applicable. LF/HF ratio: relationship between low frequency and high frequency, RMSSD: root mean square of successive differences.

**Table 5:** Indicators of body composition and athletic performance before and after the intervention for the control group (n = 15).

Indicators	Before (X ± SD)	After (X ± SD)	Variation (%)	*p*-Value	Effect Size (d)
Metabolic Equivalent of Task	2551.07 ± 386.48	2566.33 ± 407.83	0.60	0.48	0.04
Weight (kg)	63.36 ± 5.54	63.65 ± 6.43	0.46	0.61	0.05
Height (cm)	168.37 ± 5.58	168.37 ± 5.58	N/A	N/A	N/A
Body mass index (kg/m^2^)	22.36 ± 1.70	22.46 ± 2.03	0.45	0.61	0.05
Waist circumference (cm)	79.25 ± 5.66	77.37 ± 5.51	2.37	≤0.01	0.34
Left-hand grip (kg)	56.83 ± 3.98	57.11 ± 3.87	0.49	0.32	0.07
Right-hand grip (kg)	58.04 ± 4.20	60.37 ± 4.22	4.02	≤0.01	0.55
Running anaerobic sprint test (s)	4.81 ± 0,10	4.76 ± 0.11	1.04	≤0.01	0.50
Six min walking test (m)	683.01 ± 25.78	697.80 ± 32.82	2.17	≤0.01	0.50
LF/HF ratio	1.37 ± 0.17	1.30 ± 0.17	5.11	≤0.01	0.41
RMSSD (m/s)	50.41 ± 2.90	52.33 ± 3.04	3.81	≤0.01	0.65

X: mean, SD: standard deviation, N/A: not applicable. LF/HF ratio: relationship between low frequency and high frequency, RMSSD: root mean square of successive differences.

**Table 6:** Indicators of body composition and athletic performance after the intervention for the experimental group (n = 15) and control group (n = 15).

Indicators	Experimental Group (X ± SD)	Control Group (X ± SD)	Variation (%)	*p*-Value	Effect Size (d)
Metabolic Equivalent of Task	2222.87 ± 429.26	2566.33 ± 407.83	15.4	0.11	0.82
Weight (kg)	63.45 ± 5.76	63.65 ± 6.43	0.31	0.93	0.03
Height (cm)	171.38 ± 5.43	168.37 ± 5.58	1.79	0.19	0.55
Body mass index (kg/m^2^)	21.64 ± 1.97	22.46 ± 2.03	3.79	0.32	0.41
Waist circumference (cm)	80.45 ± 4.81	77.37 ± 5.51	3.95	0.10	0.60
Left-hand grip (kg)	56.0 ± 5.74	57.11 ± 3.87	1.98	0.51	0.23
Right-hand grip (kg)	58.63 ± 2.96	60.37 ± 4.22	2.97	0.15	0.48
Running anaerobic sprint test (s)	4.74 ± 0.12	4.76 ± 0.11	0.42	0.68	0.18
Six min walking test (m)	729.34 ± 46.85	697.80 ± 32.82	4.53	0.04	0.78
LF/HF ratio	1.24 ± 0.16	1.30 ± 0.17	4.84	0.37	0.36
RMSSD (m/s)	52.12 ± 5.71	52.33 ± 3.04	0.40	0.89	0.05

X: mean, SD: standard deviation. LF/HF ratio: relationship between low frequency and high frequency, RMSSD: root mean square of successive differences.

**Table 7:** ANCOVA analysis for the 6 min walk test.

Variable	Unstandardized Coefficient (B)	Standard Error	95% CI	*p*-Value
Intercept	141.11	79.75	−22.51 to 304.73	0.09
Predictive effects of pre-test on post-test	0.81	0.12	0.58 to 1.05	<0.001
Adjusted difference between groups (IBRT vs. CRT)	19.51	9.13	0.79 to 38.23	0.04
R^2^	0.69			
Adjusted R^2^	0.67			

## Data Availability

The data from this article will be made available by the authors on reasonable request.
